# Interim analyses of a first-in-human phase 1/2 mRNA trial for propionic acidaemia

**DOI:** 10.1038/s41586-024-07266-7

**Published:** 2024-04-03

**Authors:** Dwight Koeberl, Andreas Schulze, Neal Sondheimer, Gerald S. Lipshutz, Tarekegn Geberhiwot, Lerong Li, Rajnish Saini, Junxiang Luo, Vanja Sikirica, Ling Jin, Min Liang, Mary Leuchars, Stephanie Grunewald

**Affiliations:** 1 Duke University School of Medicine, Durham, NC USA; 2 Hospital for Sick Children and University of Toronto, Toronto, Ontario Canada; 3 University of California at Los Angeles (UCLA), Los Angeles, CA USA; 4 University of Birmingham, Birmingham, UK; 5 Moderna, Inc., Cambridge, MA USA; 6 Great Ormond Street Hospital for Children and Institute for Child Health, NIHR Biomedical Research Centre, London, UK

**Keywords:** Diseases, Medical research

## Abstract

Propionic acidaemia is a rare disorder caused by defects in the propionyl-coenzyme A carboxylase α or β (PCCA or PCCB) subunits that leads to an accumulation of toxic metabolites and to recurrent, life-threatening metabolic decompensation events. Here we report interim analyses of a first-in-human, phase 1/2, open-label, dose-optimization study and an extension study evaluating the safety and efficacy of mRNA-3927, a dual mRNA therapy encoding PCCA and PCCB. As of 31 May 2023, 16 participants were enrolled across 5 dose cohorts. Twelve of the 16 participants completed the dose-optimization study and enrolled in the extension study. A total of 346 intravenous doses of mRNA-3927 were administered over a total of 15.69 person-years of treatment. No dose-limiting toxicities occurred. Treatment-emergent adverse events were reported in 15 out of the 16 (93.8%) participants. Preliminary analysis suggests an increase in the exposure to mRNA-3927 with dose escalation, and a 70% reduction in the risk of metabolic decompensation events among 8 participants who reported them in the 12-month pretreatment period.

## Abstract (web summary)

Interim data from a clinical trial of mRNA-3927—an mRNA therapeutic for propionic acidaemia—provide early indications of the safety and efficacy of the treatment, and suggest that this approach might be applicable to other rare diseases.

## Main

**Fig. 1:**
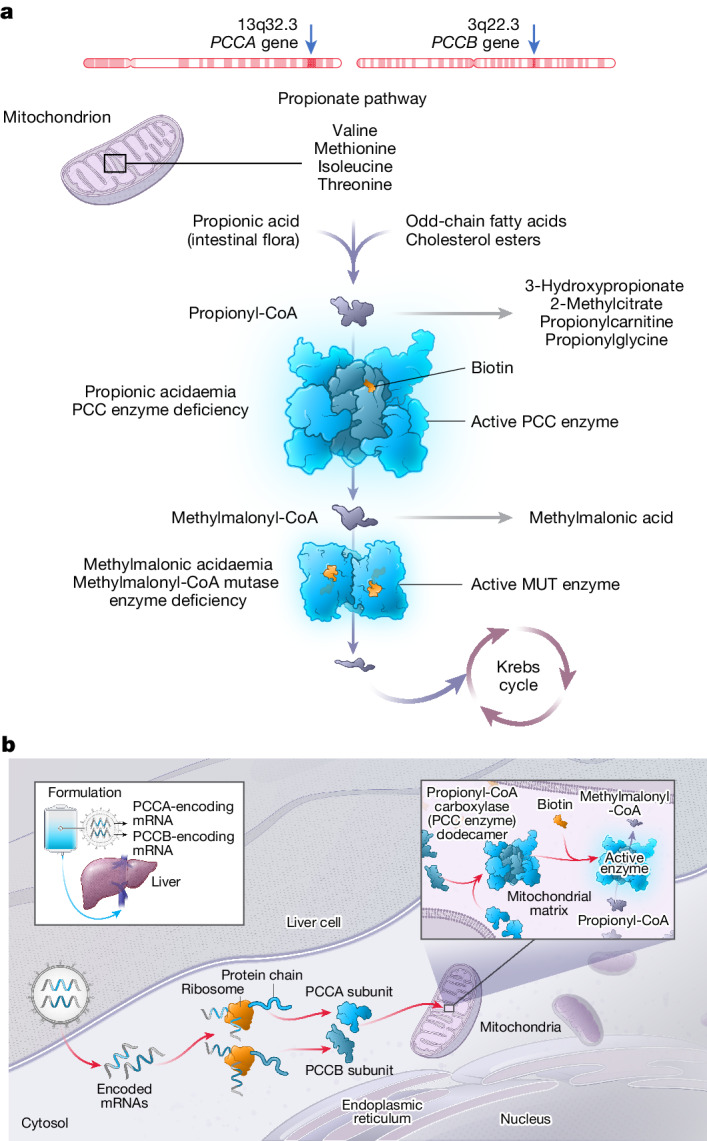
*PCCA* and *PCCB* genes, the propionate pathway and the mechanism of action of mRNA-3927. **a**, PA is caused by pathogenic variants in the *PCCA* or *PCCB* genes, resulting in a deficiency of the mitochondrial enzyme PCC. PCC deficiency leads to the accumulation of toxic metabolites, including 2-MC and 3-HP. **b**, LNP-encapsulated mRNAs encoding the human PCCA and PCCB subunits are delivered to target cells in the liver. After entry into the cytoplasm, the mRNAs are translated into the PCCA and PCCB subunits, forming the PCC enzyme complex, which localizes to the mitochondria to function in the propionate metabolic pathway. MUT, mutase. The illustration in **b** was adapted from ref. ^19^, Elsevier, under a Creative Commons licence CC BY 4.0.

Propionic acidaemia (PA; OMIM 606054) is a rare, intoxication-type, inherited metabolic disorder caused by pathogenic variants in the propionyl-coenzyme A (CoA) carboxylase (PCC) α and/or β subunit genes (*PCCA* and *PCCB*, respectively)^1^. PCC is a mitochondrial enzyme that catalyses the conversion of propionyl-CoA to methylmalonyl-CoA in the propionate metabolism pathway (Fig. 1a). Deficiencies in PCC lead to an accumulation of toxic metabolites, including propionyl-CoA and organic acids, such as 2-methylcitrate (2-MC) and 3-hydroxypropionate (3-HP)^2,3^. The birth prevalence of PA varies by country, affecting approximately 1 in 100,000 to 150,000 individuals in Western populations, with onset typically occurring during the neonatal or infantile years^4^.

PA is characterized by life-threatening metabolic decompensation events (MDEs) that often present in neonates with vomiting, dehydration, weight loss, feeding difficulties and acute deteriorations in the individual’s general clinical condition^4,5^. This presentation is often followed by progressive encephalopathy, characterized by lethargy, seizures, decreased arousal and coma, which can be disabling or fatal if not promptly treated. Multisystemic complications of PA include growth retardation, neurological manifestations, cardiomyopathy, arrythmias, recurrent pancreatitis, bone-marrow suppression and predisposition to infection^5–7^. The current guidelines for managing PA specify that survival is the most important outcome, followed by health-related quality of life, which is based on caregiver questionnaires that can capture changes in functioning^8,9^.

Early intervention is crucial to prevent metabolic decompensations, reduce mortality and morbidity and improve outcomes in those with PA; however, there are no approved drugs that directly address the underlying enzymatic defect^8^. mRNA-3927 is a novel, lipid nanoparticle (LNP)-encapsulated mRNA-based therapeutic agent composed of two mRNAs: one encoding the normal human PCCA protein subunit and the other encoding the normal human PCCB protein subunit^10^ (Fig. 1b). In addition to their mRNA payload, LNPs that are used for mRNA delivery consist of a specific assortment of lipids, including polyethylene glycol (PEG). The presence of PEG on the surface of LNPs curtails uptake of the LNP by phagocytic cells, including Kupffer cells in the liver, and thereby promotes targeted delivery of the mRNA to hepatocytes^11^. Intravenous (IV) administration of mRNA-3927 is intended to restore functional PCC enzyme in the livers of people with PA. Although nonclinical studies of LNPs have provided a proof of concept for mRNA-based therapies in mouse models of PA, such therapies have yet to be assessed in humans. Here we report the interim results of a phase 1/2 study assessing the safety and efficacy of mRNA-3927 in participants aged one year or older who have genetically confirmed PA.

## Participants

**Fig. 2:**
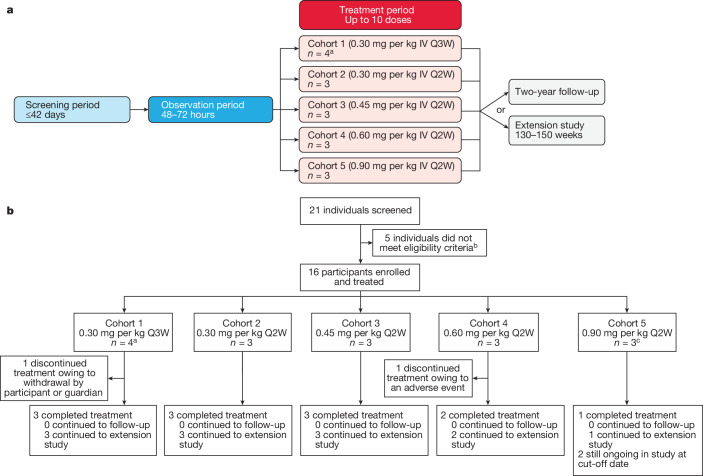
Dose-optimization study design and participant information. **a**, Study schema. Participant eligibility was assessed during the screening period, followed by an observation period during which baseline PA disease status was monitored. The enrolment of participants into each cohort was staggered using a sentinel dosing strategy. Three participants were enrolled into a cohort, treated and monitored for safety for at least 14 days by a Safety Monitoring Committee. After confirmation that no DLTs occurred, enrolment and dosing of the next cohort began. After the treatment period, participants could roll over into the extension study or enter a two-year follow-up period. Q2W, every two weeks; Q3W, every three weeks. **b**, CONSORT diagram. ^a^An additional participant was enrolled in cohort 1 per protocol after the discontinuation of one participant after their first dose owing to the withdrawal of participant consent. ^b^Seven individuals failed the initial screening; two of these individuals were rescreened and met eligibility criteria for inclusion in the study. ^c^One participant switched to 0.60 mg per kg every two weeks dosing.

Between 15 April 2021 and 9 May 2023, a total of 21 individuals were screened. As of the cut-off date of 31 May 2023, 16 participants received mRNA-3927 at 5 dose levels (cohort 1, *n* = 4; cohorts 2–5, *n* = 3 per group; Fig. 2). Three participants were initially enrolled in cohort 1; however, an extra participant was enrolled after one participant discontinued the study after their first uneventful dose owing to withdrawal of consent by the participant or guardian. A second participant (cohort 4) discontinued the study after a grade 2 infusion-site reaction (Fig. 2).

The median (range) age for all cohorts was 8.5 (1.3–26.8) years, with a median (range) age of disease onset of 0 (0–3) months (Table 1). Across all cohorts, a high proportion of participants were white (7/16; 43.8%) or Asian (5/16; 31.3%). Median (range) baseline weight ranged from 15.8 (10.6–24.8) kg in cohort 2 to 44.4 (21.6–66.5) kg in cohort 1. Across all cohorts, the most common comorbidities at baseline were developmental delay (15/16; 93.8%) and learning disorder (13/16; 81.3%).

**Table 1:** Participant demographics and baseline characteristics (intention-to-treat population)

	Cohort 1 (0.30 mg per kg Q3W; *n* = 4)	Cohort 2 (0.30 mg per kg Q2W; *n* = 3)	Cohort 3 (0.45 mg per kg Q2W; *n* = 3)	Cohort 4 (0.60 mg per kg Q2W; *n* = 3)	Cohort 5 (0.90 mg per kg Q2W; *n* = 3)	Total (*n* = 16)
Age at enrolment, years, median (range)	15.4 (5.2–26.8)	2.3 (1.5–8.3)	3.8 (1.6–15.3)	8.8 (1.3–21.4)	15.1 (1.4–17.8)	8.5 (1.3–26.8)
Age group at enrolment, years, *n* (%)						
1–2	0	2 (66.7)	1 (33.3)	1 (33.3)	1 (33.3)	5 (31.3)
3–12	2 (50.0)	1 (33.3)	1 (33.3)	1 (33.3)	0	5 (31.3)
13–17	0	0	1 (33.3)	0	2 (66.7)	3 (18.8)
≥18	2 (50.0)	0	0	1 (33.3)	0	3 (18.8)
Age at disease onset, months, median (range)	0 (0–1)	0 (0–0)	0 (0–1)	1.5 (0–3)^a^	0 (0–0)^a^	0 (0–3)
Sex, *n*						
Male:female	2:2	0:3	2:1	2:1	2:1	8:8
Race, *n* (%)						
Asian	3 (75.0)	0	1 (33.3)	0	1 (33.3)	5 (31.3)
Black or African American	0	0	1 (33.3)	0	1 (33.3)	2 (12.5)
White	1 (25.0)	2 (66.7)	1 (33.3)	2 (66.7)	1 (33.3)	7 (43.8)
Other	0	1 (33.3)	0	1 (33.3)	0	2 (12.5)
Ethnicity, *n*						
Not Hispanic or Latino	4	3	3	3	3	16
Region, *n* (%)						
Canada	2 (50.0)	0	1 (33.3)	0	1 (33.3)	4 (25.0)
UK	1 (25.0)	2 (66.7)	1 (33.3)	3 (100.0)	0	7 (43.8)
USA	1 (25.0)	1 (33.3)	1 (33.3)	0	2 (66.7)	5 (31.3)
Weight at baseline, kg, median (range)	44.4 (21.6–66.5)	15.8 (10.6–24.8)	18.0 (11.2–42.7)	24.9 (11.9–62.7)	39.3 (11.7–88.1)	24.7 (10.6–88.1)
Genotype						
*PCCA*:*PCCB*	2:2	1:2	2:1	2:1	1:2	8:8
Comorbidities^b^						
Developmental delay	3 (75.0)	3 (100.0)	3 (100.0)	3 (100.0)	3 (100.0)	15 (93.8)
Learning disorders	3 (75.0)	2 (66.7)	3 (100.0)	3 (100.0)	2 (66.7)	13 (81.3)
Encephalopathy	3 (75.0)	0	3 (100.0)	2 (66.7)	2 (66.7)	10 (62.5)
Hypotonia	3 (75.0)	2 (66.7)	2 (66.7)	1 (33.3)	2 (66.7)	10 (62.5)
Anaemia	3 (75.0)	2 (66.7)	2 (66.7)	1 (33.3)	2 (66.7)	10 (62.5)
Seizure	3 (75.0)	2 (66.7)	2 (66.7)	1 (33.3)	1 (33.3)	9 (56.3)
Neutropaenia	3 (75.0)	1 (33.3)	1 (33.3)	0	3 (100.0)	8 (50.0)
Pancytopenia	2 (50.0)	2 (66.7)	0	1 (33.3)	2 (66.7)	7 (43.8)
Thrombocytopaenia	2 (50.0)	2 (66.7)	1 (33.3)	0	1 (33.3)	6 (37.5)
Electrocardiogram QT prolonged	2 (50.0)	0	1 (33.3)	0	2 (66.7)	5 (31.3)
Pancreatitis	0	1 (33.3)	0	0	1 (33.3)	2 (12.5)
Cardiac failure	1 (25.0)	0	0	0	0	1 (6.3)
Cardiomyopathy	1 (25.0)	0	0	0	0	1 (6.3)

^a^Data missing for one participant.

^b^Preferred terms were coded using the *Medical Dictionary for Regulatory Activities* v.26.0. The comorbidities presented in the table were reported in at least five participants; in addition, the clinically significant comorbidities pancreatitis, cardiac failure and cardiomyopathy are presented.

Twelve (75.0%) participants completed the treatment period of the dose-optimization study and continued receiving study treatment in the open-label extension study. Two participants in cohort 5 (0.90 mg per kg every two weeks) were still receiving treatment in the dose-optimization study at the time of the data cut. Across both studies, a total of 346 IV doses of mRNA-3927 were administered (Extended Data Table 1) in a total of 15.69 person-years of treatment to date. Five of 16 participants who received at least one dose changed dosing regimens. Except for one participant in cohort 5 who had a dose decrease in the treatment period of the dose-optimization study (grade 3 pancreatitis and increased lipase), all dose switches occurred in the extension study, with doses increased to an amount that was considered safe and therapeutic in the dose-optimization study. The participant in cohort 5 whose dose was decreased had a history of recurrent pancreatitis, particularly associated with viral infections. After dose 1, the participant experienced a grade 3 adverse event of increased lipase that was not considered to be related to the study drug. The participant was later hospitalized for metapneumovirus; consequently, dose 2 was delayed. After dose 2, the participant experienced acute pancreatitis; this was considered by the investigator to be related to the study drug and was associated with a lipase level of 1,419 (10.9 × the upper limit of normal), but with normal imaging findings. The decision was made to reduce the participant’s dose from 0.90 mg per kg to 0.60 mg per kg. The participant skipped dose 3 and resumed dosing at 0.60 mg per kg after recovering from pancreatitis.

## Safety

No dose-limiting toxicities (DLTs) occurred. Treatment-emergent adverse events (TEAEs) were reported in 15/16 (93.8%) participants and mRNA-3927-related TEAEs were reported in 9/16 (56.3%) participants. The most common TEAEs included pyrexia (11/16; 68.8%), diarrhoea (8/16; 50.0%) and vomiting (8/16; 50.0%). TEAEs reported in more than two participants are presented in Table 2.

**Table 2:** Most common (observed in more than two participants) TEAEs (safety population)

	Cohort 1 (0.30 mg per kg Q3W)	Cohort 2 (0.30 mg per kg Q2W)	Cohort 3 (0.45 mg per kg Q2W)	Cohort 4 (0.60 mg per kg Q2W)	Cohort 5 (0.90 mg per kg Q2W)	Total
Participants initially assigned, *n*	4	3	3	3	3	16
Participants receiving at least 1 dose^a^, *n*	4	5	3	7	3	16
Participants with at least one TEAE, *n* (%)	3 (75.0)	5 (100.0)	3 (100.0)	4 (57.1)	3 (100.0)	15 (93.8)
TEAEs^b^ occurring in more than two participants overall, *n* (%)						
Pyrexia	3 (75.0)	4 (80.0)	1 (33.3)	2 (28.6)	2 (66.7)	11 (68.8)
Diarrhoea	2 (50.0)	3 (60.0)	1 (33.3)	2 (28.6)	0	8 (50.0)
Vomiting	1 (25.0)	4 (80.0)	1 (33.3)	3 (42.9)	0	8 (50.0)
Cough	1 (25.0)	2 (40.0)	1 (33.3)	2 (28.6)	1 (33.3)	6 (37.5)
Upper respiratory tract infection	2 (50.0)	3 (60.0)	1 (33.3)	1 (14.3)	0	6 (37.5)
COVID-19	1 (25.0)	3 (60.0)	0	1 (14.3)	0	5 (31.3)
Diaper dermatitis	1 (25.0)	2 (40.0)	0	2 (28.6)	0	5 (31.3)
Nasopharyngitis	0	1 (20.0)	1 (33.3)	1 (14.3)	1 (33.3)	4 (25.0)
Rhinorrhoea	0	3 (60.0)	1 (33.3)	0	0	4 (25.0)
Blood creatine phosphokinase increased	2 (50.0)	0	1 (33.3)	0	0	3 (18.8)
Ear pain	1 (25.0)	2 (40.0)	1 (33.3)	0	0	3 (18.8)
Gastroenteritis	1 (25.0)	0	0	2 (28.6)	0	3 (18.8)
Lipase increased	0	1 (20.0)	0	0	2 (66.7)	3 (18.8)
Metabolic disorder^c^	2 (50.0)	1 (20.0)	0	0	0	3 (18.8)
Rash	1 (25.0)	0	0	1 (14.3)	1 (33.3)	3 (18.8)
Rhinitis	0	1 (20.0)	1 (33.3)	1 (14.3)	0	3 (18.8)

^a^If participants changed dosing regimens, they were counted and summarized in each regimen for which they received at least one dose. The numbers were used as the denominator in the percentage calculation.

^b^TEAEs, defined as any event not present before exposure to the study drug, or any event already present that worsens in intensity or increases in frequency after exposure to the study drug, were encoded with the preferred terms of the *Medical Dictionary for Regulatory Activities* v.26.0.

^c^This was the preferred term as defined by the *Medical Dictionary for Regulatory Activities* v.26.0, used to specify an MDE; none were considered to be treatment related.

Serious adverse effects (SAEs) were reported in 8/16 (50.0%) participants. There were 2/16 (12.5%) participants who reported a total of three drug-related SAEs, including one participant with grade 3 pancreatitis and one participant with vascular device infection and injection-site reaction (both grade 2). Infusion-related reactions (IRRs) occurred in 5/16 (31.3%) participants and in 18 of the total 346 (5.2%) doses administered, including 13/73 (17.8%) doses at 0.30 mg per kg every three weeks, 0/136 (0%) doses at 0.30 mg per kg every two weeks, 1/68 (1.5%) doses at 0.45 mg per kg every two weeks, 1/45 (2.2%) doses at 0.60 mg per kg every two weeks and 3/24 (12.5%) doses at 0.90 mg per kg every two weeks. All IRRs were grade 1 or 2 in severity and generally occurred during the first few doses. All participants recovered from IRRs and continued treatment with the study drug. Two participants experienced three events of grade 1 or 2 rash.

## Antibody response

In the blood samples that were tested, one participant in cohort 1 (0.30 mg per kg every three weeks) was positive for anti-PEG antibodies at baseline. Antibody titres increased by at least fourfold in the predose sample for dose 3 (52 days after the first dose), then gradually decreased and returned to baseline levels at month 12. This participant experienced grade 1 or 2 IRRs during the infusions of 11/43 (25.6%) doses received. The participant continued receiving mRNA-3927 and has not experienced IRRs for more than a year. One participant in cohort 4 (0.60 mg per kg every two weeks) tested positive for anti-PCC antibodies at baseline but was negative at all assessments thereafter.

## PK, pharmacodynamic response, and MDE reduction

**Fig. 3:**
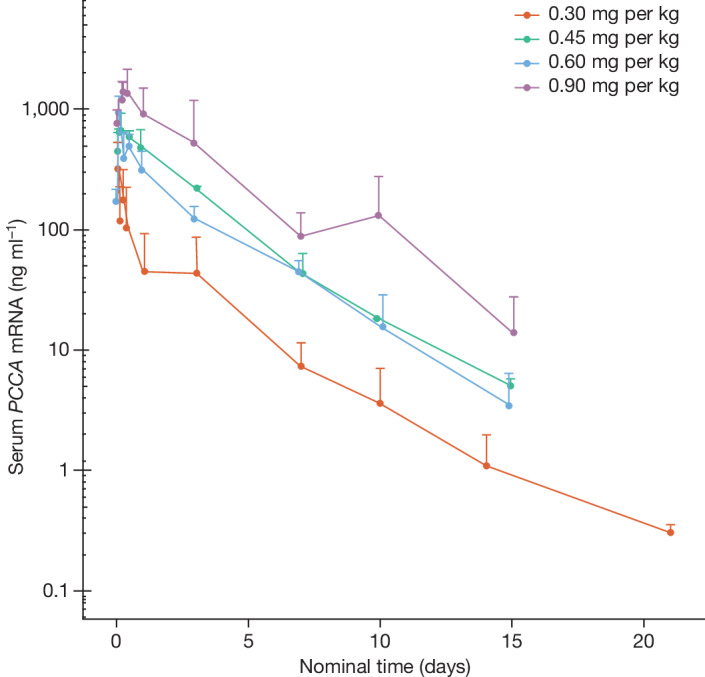
PK exposure to *PCCA* mRNA after dose 1 (PK analysis population). Blood samples were collected before dosing and at the indicated time points for up 21 days after dose 1. Samples were submitted to the PPD central laboratory for quantification of *PCCA* mRNA through branched DNA analysis. For graphing purposes, cohorts 1 and 2 (both 0.30 mg per kg) were combined before the dosing frequency changed for dose 2. Data are mean + s.d.

The pharmacokinetic (PK) parameters of *PCCA* and *PCCB* mRNA after a single dose and repeated doses were assessed in available cohorts (through cohort 5) at the time of the data cut. Overall, the PK exposure to *PCCA* mRNA increased with dose escalation; *PCCB* testing is still ongoing. PK exposures to *PCCA* mRNA for all available cohorts after dose 1 are shown in Fig. 3 and Extended Data Table 2.

Reductions from baseline in disease biomarkers, including 3-HP, 2-MC, propionylcarnitine (C3) and *n*-propionylglycine (n-PG), were observed after dose 3 with mRNA-3927 in most patients (Extended Data Table 3).

**Fig. 4:**
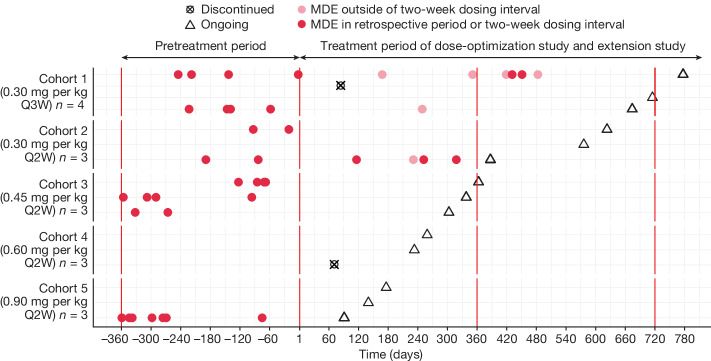
MDE profiles (intention-to-treat population). MDEs, defined as the presence of exacerbated PA symptoms, the need for emergency care and evidence of biochemical abnormalities, were assessed 12 months before treatment and in the post-treatment period. Note that the definition of MDEs was updated during the study in response to discussions with regulatory agencies. MDEs that were reported by investigators before this update might not fully align with this current definition.

Clinical efficacy was evaluated on the basis of the frequency of MDEs, defined as an exacerbation of PA-related symptoms with biochemical abnormalities requiring emergency care or hospitalization. Overall, 8/16 (50.0%) participants experienced one or more MDEs during the pretreatment period, 2/16 (12.5%) during the treatment period of the dose-optimization study and 3/16 (18.8%) during the treatment period of the dose-optimization study and the extension study (Fig. 4 and Extended Data Table 4). No MDEs were observed in cohort 4 (0.60 mg per kg every two weeks) in the pretreatment or treatment periods of the dose-optimization study before the last follow-up. Across all cohorts in the combined dose-optimization and extension studies, participants had an overall relative risk of MDEs of 0.30 (95% confidence interval (CI), 0.066–1.315) during the treatment period compared to the pretreatment period (*P* = 0.0927). Among only the dosing cohorts receiving treatment every two weeks, the overall relative risk of MDEs in the treatment period versus the pretreatment period was 0.18 (95% CI, 0.011–2.907; *P* = 0.1832).

## Discussion

To our knowledge, this first-in-human dose-optimization study is the first clinical trial reporting the results of an mRNA therapeutic for intracellular protein replacement in an ultra-rare disease for which there is a considerable unmet need for treatment options. As of the 31 May 2023 data cut, 16 participants had received mRNA-3927, with more than 340 IV doses administered. Two participants discontinued the study, and two participants are still undergoing treatment in the dose-optimization study; all others are continuing treatment in the extension study.

So far, mRNA-3927 has been well tolerated in participants with PA at the doses administered, with no DLTs observed, including the adverse event of pancreatitis described, which was assessed as related to PA by the Safety Monitoring Committee and the sponsor. As an adverse event of special interest, IRRs are common among individuals who are receiving IV therapy^12^. Herein, IRRs occurred in 31.3% of participants and 5.2% of the doses administered, one of which resulted in the withdrawal of one participant. A similar incidence of IRRs has been reported in clinical studies evaluating IV recombinant enzyme replacement therapy for other rare diseases^13^. In our study, participants received premedication with paracetamol or ibuprofen and histamine receptor (H_1_R and H_2_R) blockers before administration of the study drug, to reduce the possibility of IRRs. IRRs that emerged during the infusion were managed by slowing the infusion rate and/or by a combination of antipyretics and corticosteroids according to established methods^14^. Treatment with glucocorticoids was well tolerated and not associated with MDEs.

In this interim analysis, a reduction in the number of MDEs was observed for all except two participants (both in subtherapeutic dose cohorts), who had reported MDEs in the pretreatment period. The relative risk for MDEs was reduced by 70% during the treatment period of the dose-optimization study and by 82% among the biweekly treatment cohorts. Furthermore, although early in the observation period at the time of the data cut, no MDEs were observed with higher doses. By comparison, the incidence of metabolic episodes after liver transplantation—a treatment option for patients with PA to achieve metabolic stability—has been highly variable, and any benefit in metabolic stability gained from liver transplantation must be balanced with the chance of surgical complications and the need for lifelong immunosuppression after the transplant^15^.

In this ongoing study, the PK parameters of *PCCA* and *PCCB* mRNA after a single dose and repeated doses were assessed as secondary endpoints. The available data for *PCCA* mRNA suggest a trend towards increased PK exposure to mRNA-3927 with dose escalation. Several disease biomarkers were also monitored, including 3-HP, 2-MC, C3 and n-PG. On the basis of health-care guidelines^8^, biochemical metabolite levels (such as 2-MC and C3) are often used as surrogate markers for metabolic stability^8^. In the ongoing study, most patients showed reductions in all four of the above-mentioned biomarkers after treatment with mRNA-3927. Together with reductions in MDEs, these data suggest that IV infusion of mRNA-3927 resulted in successful transportation into liver cells, protein translation and post-translational modification into the active PCC enzyme.

Although this study represents a first-in-human investigation into the safety and efficacy of LNP treatment for a rare disease, it is not without limitations. These include a small sample size for each cohort, the absence of a control group, the open-label nature of the study design and the shorter observation times for the higher-dose cohorts compared with the lower-dose cohorts. Although appropriate and necessary for a first-in-human phase 1 study, the small sample size precludes the possibility of performing statistical analyses to assess the significance of these findings. Moreover, the lack of a control group might yield higher estimates of mean effects, possibly inflating the efficacy of the intervention. In addition, only half of the participants (8/16) assessed experienced an MDE before treatment, thereby limiting the subset available to fully assess therapeutic efficacy as measured by MDE reduction. Finally, with the limited amount of data pertaining to LNP-delivered therapeutics in humans, drawing conclusions on the basis of cross-trial comparisons is difficult. However, these results are in line with previously published reports that have shown that therapeutics, including interfering RNAs and mRNA vaccines formulated in LNPs, are safe and effective^16–18^. Given that different diseases have unique biological mechanisms, tissue and organ specificities and populations of individuals who are affected, the findings reported here might not be generalizable to all replacement therapies. However, these results could be particularly applicable to diseases in which the protein being replaced both is expressed in and functions in the liver, which is true not only for PA, but also for other metabolic disorders, including methylmalonic acidaemia.

This study is ongoing, and participants in cohort 5 (0.90 mg per kg every two weeks) are still being assessed. Once the cohort 5 analysis is complete, the optimal therapeutic dose may be identified, and the dose-expansion portion of the study will commence. For the dose expansion, up to 15 participants will receive mRNA-3927 for 12 months at the selected dose to evaluate the efficacy of the optimal dose and to further characterize its safety, tolerability, efficacy and pharmacological activity in participants with PA.

In conclusion, these interim results are encouraging and show that mRNA-3927 could be of clinical benefit for patients with PA.

## Methods

### Trial design and oversight

PARAMOUNT (ClinicalTrials.gov identifier: NCT04159103) is an ongoing, phase 1/2, international, open-label, multicentre, dose-optimization (part 1) and dose-expansion (part 2) study assessing the safety, pharmacokinetics and efficacy of mRNA-3927. The methods and interim results pertaining to the part 1 dose-optimization study are reported herein. The trial was conducted at 15 centres in 3 countries (Canada, the UK and the USA) and was sponsored by Moderna. The study was conducted in accordance with consensus ethical principles and regulatory guidelines including the Declaration of Helsinki, the Council for International Organizations of Medical Sciences International Ethical Guidelines and the International Council of Harmonisation Guidelines on Good Clinical Practice (ICH-GCP). The final trial protocol and amendments were approved by an independent ethics committee, research ethics board or institutional review board at the participating sites. All participants and/or a legally authorized representative provided written informed consent.

### Participants

Individuals who were at least one year of age at the time of consent or assent and who had a confirmed diagnosis of PA based on molecular genetic testing (*PCCA* and/or *PCCB* mutations) were eligible. The first two participants to enrol were required to be at least eight years of age. Participants with childbearing potential agreed to use a highly effective method of contraception during study treatment and for three months after the last administration of the study drug. Participants were excluded in the following cases: laboratory abnormalities exceeding exclusionary thresholds (Supplementary Table 1); estimated glomerular filtration rate less than 30 ml per min per 1.73 m^2^; chronic dialysis; QTc greater than 480 ms using Bazett’s correction; positive pregnancy test or pregnant or breastfeeding; grade 3 or 4 heart failure; history of or planned organ transplant; hypersensitivity to premedications or drug components; a major surgical procedure within 30 days (excluding a central line, port or feeding tube); COVID-19 vaccination within 6 weeks between the last dose and the first administration of the study drug; or on another investigational agent within 30 days or within 5 elimination half-lives.

### mRNA-3927 production and formulation

mRNA was synthesized and LNPs were formulated as previously described^10,19^. In brief, in vitro, T7 RNA polymerase-mediated transcription was used to generate codon-optimized mRNAs encoding human PCCA (hPCCA) and human PCCB (hPCCB) proteins. LNPs consisted of a mixture of four lipids: SM-86, OL-56 (a PEG-lipid conjugate), distearoylphosphatidylcholine and cholesterol. mRNA was mixed with lipids at a 3:1 molar ratio. Each mRNA-3927 IV unit contained 2.0 mg ml^−1^ of total RNA (hPCCA and hPCCB subunits at a 1:1 molar ratio) in the LNP dispersion formulated in 20 mM Tris (pH 7.5), 8% sucrose and 1 mM diethylenetriaminepentaacetic acid. Formulated LNPs were 87 nm in diameter, had a polydispersity index of 0.2, were more than 96% encapsulated and had less than 2 EU ml^−1^ endotoxin. mRNA-3927 infusions were prepared in glass vials at a final volume of 2.8 ml and were stored at −60 °C to −90 °C.

### Trial procedures

Participants were allocated by nonrandom assignment. Participants were enrolled into five cohorts with staggered enrolment and dosing (Fig. 2). Eligibility was first determined during the screening period (42 days maximum), after which participants entered the observation period (48–72 h), followed by the treatment period (up to ten doses over 20–40 weeks dependent on the dosing schedule). Participants could then choose to enter a two-year safety follow-up period or continue to receive mRNA-3927 in an open-label extension study (ClinicalTrials.gov identifier: NCT05130437).

During the screening period, PA diagnosis was confirmed, and the participant’s medical history and health status were collected (Fig. 2). Baseline PA disease status was monitored during the observation period, during which time blood biomarkers (2-MC, 3-HP, C3 and n-PG) and antibody status (anti-PEG and anti-PCC) were assessed. Before treatment (60 ± 10 min), participants received paracetamol or ibuprofen, with age- and/or weight-appropriate dosing, and a histamine receptor (H_1_R and H_2_R) blocker.

During the treatment period, participants received the specified dose of mRNA-3927 (mg per kg), which was based on mRNA concentrations only and did not consider the molecular weight of the LNP. IV infusions occurred over 1 to 6 h (±15 min). After treatment, participants were discharged only after resolution of any IRRs, no evidence of haemodynamic or respiratory instability, resolution of any mental status changes or lightheadedness that may have occurred following the start of study-drug infusion, a return to ambulation status before dose administration (if applicable) and normal vital signs for age or return to predose values. If an IRR occurred during study-drug infusion, antipyretics or steroids were used for management according to the judgement of the investigator. Anaphylaxis was assessed and managed according to the National Institution of Allergy and Infectious Disease and Food Allergy and Anaphylaxis Network guidelines^20,21^.

During the treatment period of the dose-optimization study, the first participant in cohort 1 received 0.30 mg per kg IV mRNA-3927 every three weeks and was observed for DLTs, defined as TEAEs of grade 3 or higher, related to the study drug, that occurred within a 21-day window after the first administration of mRNA-3927. If no DLTs were observed after the 21-day window, the next participant in the cohort was treated. Once the DLT window was completed for the last participant in each cohort, the next cohort was enrolled, and treatment was initiated (cohort 2: 0.30 mg per kg every two weeks; cohort 3: 0.45 mg per kg every two weeks; cohort 4: 0.60 mg per kg every two weeks; and cohort 5: 0.90 mg per kg every two weeks). The DLT window was 14 days starting in cohort 3. After the treatment period of the dose-optimization study, participants who continued into the extension study could have their dose adjusted to the level deemed safe and efficacious in the dose-optimization study.

During the treatment period (day 1, dose 1 through to the end-of-treatment visit), participant assessments (as inpatients, outpatients, and/or through home health-care visits) included adverse events, concomitant medications and procedures, vital signs and physical examinations, PK sampling and safety laboratory assessments. If a participant missed three doses of the study drug, the sponsor could recommend discontinuation of participation in the study in consultation with the investigator. If treatment resumed, the participant continued from the point they started missing doses (for example, if a participant missed doses 3, 4 and 5 and then resumed the study drug, they resumed with dose 6).

### Assessments

The primary endpoint was the incidence and severity of TEAEs (including study-drug-related TEAEs) and SAEs and TEAEs leading to treatment discontinuation. TEAEs were defined as any adverse event with an onset date on or after the study-drug start date, including throughout the follow-up period. SAEs (TEAEs resulting in death; considered life-threatening; requiring hospitalization or prolongation of existing hospitalization; judged as medically important or resulting in persistent disability or incapacity; or congenital anomaly or birth defect), TEAEs related to the study drug and TEAEs leading to discontinuation were collected throughout the study. Along with TEAEs, adverse events of special interest for close monitoring in this study included IRRs (reactions assessed as related to the study drug or IV infusion that occurred within 24 h of treatment) and hypersensitivity reactions. The severity of all adverse events was assessed according to the Common Terminology Criteria for Adverse Events, v.5.0.

Secondary endpoints included changes in blood 2-MC and 3-HP levels from baseline (predose levels) to post-dose levels, the assessment of antibodies to PEG (anti-drug antibodies) and PK parameters of *PCCA* and *PCCB* mRNAs after single and repeated administrations of mRNA-3927. Exploratory analysis included changes from baseline (predose levels) to post-dose levels in other blood biomarkers (including C3 and n-PG), the assessment of antibodies to PCC (anti-drug antibodies) and MDEs in the pretreatment (12 months before informed consent until the first dose of treatment) and post-treatment periods. Blood samples for PK and antibody assessments were collected before and throughout the treatment period using a PPD collection kit according to v.3 of the Central Lab Manual and sent to PPD for analyses (Wilmington, NC). Antibody titres reflect all anti-PEG isotypes. *PCCA* mRNA was quantified by PPD using branched DNA^22^ analyses and PK parameters (maximum concentration observed, area under the concentration–time curve extrapolated to infinite time, clearance, terminal volume of distribution, terminal elimination half-life and time to maximum observed concentration) were assessed. In response to expert and regulatory feedback, the definition of an MDE was updated during the study. In the latest version of the protocol, an MDE was defined as the presence of exacerbated PA symptoms (persistent vomiting; anorexia or failure to feed; lethargy; change in behaviour or level of consciousness; or increased seizure activity), the need for emergency care (hospitalization or requiring at least 24 h in an emergency care unit) and evidence of biochemical abnormalities, as demonstrated by one or more of the following: acute hyperammonaemia, metabolic acidosis (decreased pH) with high anion gap or compensated metabolic acidosis (reduced bicarbonate, base deficit, reduced PaCO_2_ or increased lactate) with high anion gap. Owing to the change in definition, some MDEs reported before the protocol update may not fully align with the latest MDE definition. Other secondary outcomes, including changes in biomarker concentrations and health-related quality of life, were also assessed and are planned to be reported later with additional participants and a longer follow-up time.

An independent Safety Monitoring Committee was implemented to monitor the conduct of the study and provide an ongoing review of clinical safety and any relevant data; to recommend dose escalation after each dose cohort; or to pause or stop the study if major safety concerns emerged.

### Statistical analysis

This was not a hypothesis-based study, and no formal statistical calculation of sample size was performed. Baseline demographics were assessed in the intention-to-treat population (all enrolled participants). All safety and exposure analyses were performed in the safety population (participants who received at least one dose of mRNA-3927) and were coded according to the *Medical Dictionary for Regulatory Activities* v.26.0. If a participant changed dosing regimens, they were counted and summarized in each regimen in which they received at least one dose of mRNA-3927. Safety analyses are presented as counts and percentages of TEAEs after treatment.

PK analyses were performed in participants in the safety population who had evaluable mRNA concentrations and did not have any major protocol deviations affecting the PK assessments. Serum mRNA concentrations were summarized descriptively by nominal time point, dose level and visit. Anti-drug antibody assessment was performed in the immunogenicity population, which included members of the safety population who had at least one post-dose, evaluable anti-PEG or anti-PCC sample. A data analysis was performed by the study sponsor using SAS (v.9.4 or higher).

### Reporting summary

Further information on research design is available in the Nature Portfolio Reporting Summary linked to this article.

## Online content

Any methods, additional references, Nature Portfolio reporting summaries, source data, extended data, supplementary information, acknowledgements, peer review information; details of author contributions and competing interests; and statements of data and code availability are available at 10.1038/s41586-024-07266-7.

### Supplementary information

Supplementary Table 1

Reporting Summary

## Figures and Tables

**Extended Data Table 1:**
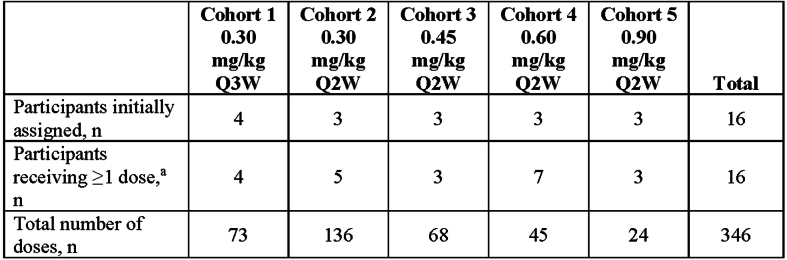
Participant exposure (safety population)

Q2W, every two weeks; Q3W, every three weeks.

^a^If participants changed dosing regimens, they were counted and summarized in each regimen for which they received at least one dose. In the extension study, two participants in cohort 1 changed to the 0.30 mg per kg Q2W dosing regimen, two participants in Cohort 2 changed to the 0.60 mg per kg Q2W dosing regimen and one participant in cohort 5 changed to the 0.60 mg per kg Q2W dosing regimen.

**Extended Data Table 2:**
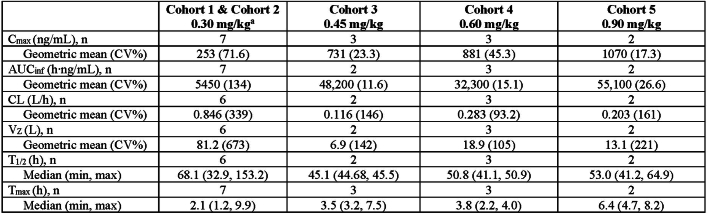
PK parameter summary statistics of serum *PCCA* mRNA after dose 1 (PK analysis population)

AUC_inf_, area under the concentration–time curve extrapolated to infinite time; CL, clearance; *C*_max_, maximum concentration observed; CV%, percent coefficient of variation; *T*_1/2_, terminal elimination half-life; *T*_max_, time to maximum observed concentration; *V*_z_, terminal volume of distribution.

^a^Because the table represents PK parameters after dose 1, cohorts 1 and 2, both of which received 0.30 mg per kg, were combined for these analyses.

**Extended Data Table 3:**
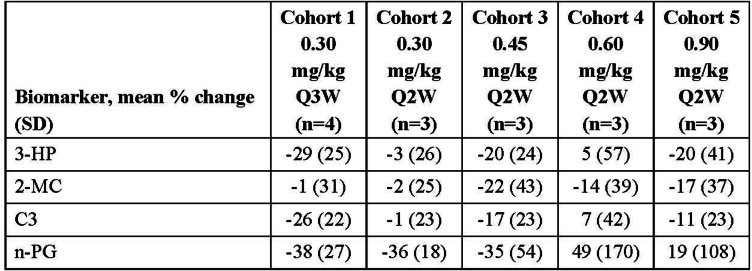
Biomarker percentage change from baseline after the third dose

Q2W, every two weeks; Q3W, every three weeks; SD, standard deviation.

**Extended Data Table 4:**
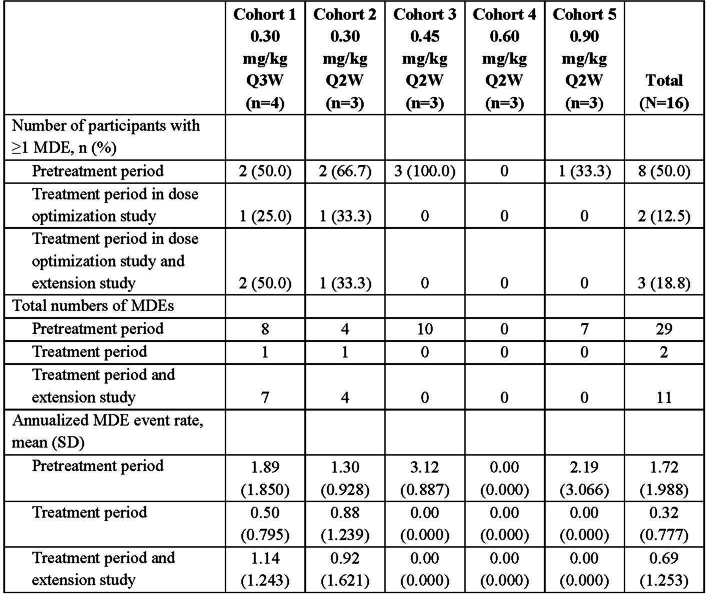
Summary of MDEs (intention-to-treat population)

Q2W, every 2 weeks; Q3W, every three weeks; SD, standard deviation.

## Data Availability

Access to patient-level data and supporting clinical documents with qualified external researchers may be available upon request and subject to review once the trial is complete.
