# Publisher Correction: Interim analyses of a first-in-human phase 1/2 mRNA trial for propionic acidaemia

**DOI:** 10.1038/s41586-024-07646-z

**Published:** 2024-06-06

**Authors:** Dwight Koeberl, Andreas Schulze, Neal Sondheimer, Gerald S. Lipshutz, Tarekegn Geberhiwot, Lerong Li, Rajnish Saini, Junxiang Luo, Vanja Sikirica, Ling Jin, Min Liang, Mary Leuchars, Stephanie Grunewald

**Affiliations:** 1 Duke University School of Medicine, Durham, NC USA; 2 Hospital for Sick Children and University of Toronto, Toronto, Ontario Canada; 3 University of California at Los Angeles (UCLA), Los Angeles, CA USA; 4 University of Birmingham, Birmingham, UK; 5 Moderna, Inc., Cambridge, MA USA; 6 Great Ormond Street Hospital for Children and Institute for Child Health, NIHR Biomedical Research Centre, London, UK

**Keywords:** Diseases, Medical research

Correction to: *Nature* 10.1038/s41586-024-07266-7 Published online 3 April 2024

This paper was originally published under standard Springer Nature license (© The Author(s), under exclusive licence to Springer Nature Limited). It is now available as an Open Access paper under a Creative Commons Attribution 4.0 International license, © The Author(s). The error has been corrected in the HTML and PDF versions of the article.

