# Supplementary material for: Interim analyses of a first-in-human phase 1/2 mRNA trial for propionic acidaemia
Source: Nature. 2024 Apr 3;628(8009):872–7. doi: 10.1038/s41586-024-07266-7 (PMC11156579; doi:10.1038/s41586-024-07266-7)
Supplement: Supplementary file 1 — Supplementary Table 1 [file 41586_2024_7266_MOESM1_ESM.pdf]

nature portfolio

https://doi.org/10.1038/s41586-024-07266-7

Supplementary information

Interim analyses of a first-in-human phase 1/2 mRNA trial for propionic acidaemia

In the format provided by the authors and unedited

nature portfolio

https://doi.org/10.1038/s41586-024-07266-7

Supplementary information

Interim analyses of a first-in-human phase 1/2 mRNA trial for propionic acidaemia

In the format provided by the authors and unedited

Supplementary Information

mRNA-3927 Therapy for Propionic Acidemia: Interim Analysis From the First-in-human, Open-label, Nonrandomized, Phase 1/2 PARAMOUNT Study

Koeberl et al

Supplementary Table 1. Protocol-required laboratory assessments.

**Laboratory assessments**	**Parameters**	**Value exclusionary for study participation**
**Laboratory safety assessments**		
Hematology	Platelet count	<75,000 mm^3
	Red blood cell count	Not exclusionary
	Hemoglobin	<9 g/dL
	Hematocrit	Not exclusionary
	RBC indices:<br>mean corpuscular volume,<br>mean corpuscular hemoglobin	Not exclusionary
	Percent reticulocytes	Not exclusionary
	White blood cell count	<2000 mm^3
	Neutrophil count	<1000 mm^3
	Neutrophil percent	Not exclusionary
	Lymphocyte count/percent	<800 mm^3
	Monocyte, eosinophil, basophil count/percent	Not exclusionary
Comprehensive metabolic panel$^+$ (CMP$^+$, with amylase and lipase)	Blood urea nitrogen	Not exclusionary
	Potassium	>5.5-6.0 mmol/L or symptomatic and <3.0 mmol/L
	Carbon dioxide (bicarbonate)	Not exclusionary
	Sodium	<130 mmol/L and symptomatic or >150 mmol/L and intervention initiated
	Chloride	Not exclusionary
	Creatinine	Not exclusionary (estimated glomerular filtration rate is evaluated instead)
	Glucose (nonfasting)	Not exclusionary
	Anion gap	Not exclusionary
	Calcium	Corrected serum calcium <8.0 mg/dL and symptomatic, or >11.5 mg/dL and symptomatic
	Aspartate aminotransferase	>2.0 × ULN
	Alanine aminotransferase	>2.0 × ULN
	Total protein	Not exclusionary
	Albumin	<3.0 g/dL
	Globulin, albumin/globulin ratio	Not exclusionary
	Gamma-glutamyl transferase	Not exclusionary
	Total bilirubin	>1.5 × ULN
	Direct bilirubin	>1.5 × ULN
	Alkaline phosphatase	>2.0 × ULN

**Laboratory assessments**	**Parameters**	**Value exclusionary for study participation**
	Lactate dehydrogenase	>1.5 × ULN
	Amylase	>1.5 × ULN and symptomatic or >2.5 × ULN and asymptomatic
	Lipase	>1.5 × ULN and symptomatic or >2.0 × ULN and asymptomatic
	Uric acid	Not exclusionary
	C-reactive protein	Not exclusionary
	Phosphorus	Not exclusionary
	Bicarbonate	Not exclusionary
	Creatinine phosphokinase	Not exclusionary
	Total cholesterol	Not exclusionary
	Triglycerides	Not exclusionary
Coagulation	Prothrombin time	>1.2 × ULN
	Activated partial thromboplastin time	>1.5 × ULN
**Other safety assessments**		
Pregnancy test (*performed at local laboratory*)	β-hCG pregnancy test (as needed for women of childbearing potential)	Positive result

ULN, upper limit of normal.
