# Supplementary material for: Interim analyses of a first-in-human phase 1/2 mRNA trial for propionic acidaemia
Source: Nature. 2024 Apr 3;628(8009):872–7. doi: 10.1038/s41586-024-07266-7 (PMC11156579; doi:10.1038/s41586-024-07266-7)
Supplement: Supplementary file 2 — Reporting Summary [file 41586_2024_7266_MOESM2_ESM.pdf]

nature portfolio

Corresponding author(s): Stephanie Grunewald, MD

Last updated by author(s): February 14, 2024

Reporting Summary

Nature Portfolio wishes to improve the reproducibility of the work that we publish. This form provides structure for consistency and transparency in reporting. For further information on Nature Portfolio policies, see our Editorial Policies and the Editorial Policy Checklist.

Statistics

For all statistical analyses, confirm that the following items are present in the figure legend, table legend, main text, or Methods section.

n/a	Confirmed	
☐	☒	The exact sample size (n) for each experimental group/condition, given as a discrete number and unit of measurement
☒	☐	A statement on whether measurements were taken from distinct samples or whether the same sample was measured repeatedly
☒	☐	The statistical test(s) used AND whether they are one- or two-sided<br>*Only common tests should be described solely by name; describe more complex techniques in the Methods section.*
☒	☐	A description of all covariates tested
☒	☐	A description of any assumptions or corrections, such as tests of normality and adjustment for multiple comparisons
☐	☒	A full description of the statistical parameters including central tendency (e.g. means) or other basic estimates (e.g. regression coefficient) AND variation (e.g. standard deviation) or associated estimates of uncertainty (e.g. confidence intervals)
☒	☐	For null hypothesis testing, the test statistic (e.g. F, t, r) with confidence intervals, effect sizes, degrees of freedom and P value noted<br>*Give P values as exact values whenever suitable.*
☒	☐	For Bayesian analysis, information on the choice of priors and Markov chain Monte Carlo settings
☒	☐	For hierarchical and complex designs, identification of the appropriate level for tests and full reporting of outcomes
☒	☐	Estimates of effect sizes (e.g. Cohen's d, Pearson's r), indicating how they were calculated

Our web collection on statistics for biologists contains articles on many of the points above.

Software and code

Policy information about availability of computer code

Data collection	*Provide a description of all commercial, open source and custom code used to collect the data in this study, specifying the version used OR state that no software was used.*
Data analysis	Data analysis was performed using SAS (Version 9.4 or higher).

For manuscripts utilizing custom algorithms or software that are central to the research but not yet described in published literature, software must be made available to editors and reviewers. We strongly encourage code deposition in a community repository (e.g. GitHub). See the Nature Portfolio guidelines for submitting code & software for further information.

Data

Policy information about availability of data

All manuscripts must include a data availability statement. This statement should provide the following information, where applicable:

- Accession codes, unique identifiers, or web links for publicly available datasets
- A description of any restrictions on data availability
- For clinical datasets or third party data, please ensure that the statement adheres to our policy

Access to patient-level data and supporting clinical documents with qualified external researchers may be available upon request and subject to review once the trial is complete

Research involving human participants, their data, or biological material

Policy information about studies with human participants or human data. See also policy information about sex, gender (identity/presentation), and sexual orientation and race, ethnicity and racism.

Reporting on sex and gender	Detailed cohort characteristics, including participant sex, is included in Table 1. Sub-analyses by sex was not conducted due to a the small sample size and the fact that natural history studies have indicated that sex does not play a role in the disease.
Reporting on race, ethnicity, or other socially relevant groupings	Detailed cohort characteristics, including participant race and ethnicity, are included in Table 1. Sub-analyses by race and ethnicity was not conducted due to the small sample size and the fact that this was beyond the scope of the study.
Population characteristics	Individuals ≥1 year of age at the time of consent/assent with a confirmed diagnosis of PA based on molecular genetic testing (PCCA and/or PCCB mutations) were eligible. The first 2 participants to enroll were required to be ≥8 years of age. Participants with childbearing potential agreed to use a highly effective method of contraception during study treatment and for 3 months following the last administration of study drug. Detailed cohort characteristics are provided in Table 1.
Recruitment	Patients were recruited for participation in 3 countries (Canada, the United Kingdom, and the United States). 16 participants received mRNA-3927 at 5 dose levels (Cohort 1: n=4; Cohorts 2-5: n=3 per group).
Ethics oversight	The study was conducted in accordance with consensus ethical principles and regulatory guidelines including the Declaration of Helsinki and Council for International Organizations of Medical Sciences International Ethical Guidelines and the International Council of Harmonisation Guidelines on Good Clinical Practice (ICH-GCP). The final trial protocol and amendments were approved by an independent ethics committee, research ethics board, or institutional review board at participating sites. All participants and/or a legally authorized representative provided written informed consent.

Note that full information on the approval of the study protocol must also be provided in the manuscript.

Field-specific reporting

Please select the one below that is the best fit for your research. If you are not sure, read the appropriate sections before making your selection.

☒ Life sciences ☐ Behavioural & social sciences ☐ Ecological, evolutionary & environmental sciences

For a reference copy of the document with all sections, see nature.com/documents/nr-reporting-summary-flat.pdf

Life sciences study design

All studies must disclose on these points even when the disclosure is negative.

Sample size	This was not a hypothesis-based study, and no formal statistical calculation of sample size was performed
Data exclusions	Five participants failed to meet inclusion criteria. Safety and exposure analyses were performed in all participants who received ≥1 dose of mRNA-3927. PK analysis was performed in participants in the safety population who had evaluable mRNA concentrations and did not have any major protocol deviations impacting the PK assessments. Assessment of serum mRNA concentrations was performed in the immunogenicity population, which included participants of the safety population who had ≥1 postdose, evaluable anti-PEG or anti-PCC sample.
Replication	Trial was only performed once without replication.
Randomization	This study was nonrandomized.
Blinding	This study was open-label.

Reporting for specific materials, systems and methods

We require information from authors about some types of materials, experimental systems and methods used in many studies. Here, indicate whether each material, system or method listed is relevant to your study. If you are not sure if a list item applies to your research, read the appropriate section before selecting a response.

Materials & experimental systems

n/a	Involved in the study
☒	☐ Antibodies
☒	☐ Eukaryotic cell lines
☒	☐ Palaeontology and archaeology
☒	☐ Animals and other organisms
☐	☒ Clinical data
☒	☐ Dual use research of concern
☒	☐ Plants

Methods

n/a	Involved in the study
☒	☐ ChIP-seq
☒	☐ Flow cytometry
☒	☐ MRI-based neuroimaging

Clinical data

Policy information about clinical studies

All manuscripts should comply with the ICMJE guidelines for publication of clinical research and a completed CONSORT checklist must be included with all submissions.

Clinical trial registration	ClinicalTrials.gov Identifier: NCT05130437
Study protocol	The study protocol will be provided
Data collection	Individuals were screened between April 15, 2021, and May 9, 2023. The cutoff date for this interim analysis is May 31, 2023.
Outcomes	The primary endpoint was the incidence and severity of TEAEs (including study drug–related TEAEs), SAEs, and TEAEs leading to treatment discontinuation. Secondary endpoints included changes in blood 2-MC and 3-HP levels from baseline (predose levels) to postdose levels, the assessment of antibodies to PEG (anti–drug antibodies), and PK parameters of PCCA and PCCB mRNAs after single and repeated administrations of mRNA-3927. Exploratory analysis included changes from baseline (predose levels) to postdose levels in other blood biomarkers (including C3 and n-PG), the assessment of antibodies to PCC (anti–drug antibodies), and MDEs in the pretreatment (12-month prior to informed consent until the first dose of treatment) and posttreatment periods. Detailed regarding how primary and secondary outcomes were assessed is outlined in the methods section of this manuscript.
